# On Linseed Oil in Hemorrhoids

**Published:** 1851-01

**Authors:** 


*On Linseed Oil in Hemorrhoids.*—Van Rye believes, that, in general, surgical treatment is too hastily resorted to in this affection, and he wishes to bring under the notice of the profession a remedy he has found of great efficacy during twenty-five years. It consists in the administration of two ounces of fresh linseed oil every morning and evening; and so rapid is the amendment generally, that the remedy is seldom continued longer than a week. Sometimes the stools are somewhat increased in quantity, but neither vomiting nor any other ill effect produced. The only precaution the while, is the abstinence from alcoholic drinks and too stimulating a diet.—*Ibid*, from *L'Union Médicale.*—*Ibid.*

